# Decoding disease-causing mechanisms of missense mutations from supramolecular structures

**DOI:** 10.1038/s41598-017-08902-1

**Published:** 2017-08-17

**Authors:** Atsushi Hijikata, Toshiyuki Tsuji, Masafumi Shionyu, Tsuyoshi Shirai

**Affiliations:** 1 Faculty of Bioscience, Nagahama Institute of Bio-Science and Technology, 1266 Tamura-cho, Nagahama, Shiga, 526-0829 Japan; 2 MITA International School, Yoga, Setagaya, Tokyo, Japan

## Abstract

The inheritance modes of pathogenic missense mutations are known to be highly associated with protein structures; recessive mutations are mainly observed in the buried region of protein structures, whereas dominant mutations are significantly enriched in the interfaces of molecular interactions. However, the differences in phenotypic impacts among various dominant mutations observed in individuals are not fully understood. In the present study, the functional effects of pathogenic missense mutations on three-dimensional macromolecular complex structures were explored in terms of dominant mutation types, namely, haploinsufficiency, dominant-negative, or toxic gain-of-function. The major types of dominant mutation were significantly associated with the different types of molecular interactions, such as protein-DNA, homo-oligomerization, or intramolecular domain-domain interactions, affected by mutations. The dominant-negative mutations were biased toward molecular interfaces for cognate protein or DNA. The haploinsufficiency mutations were enriched on the DNA interfaces. The gain-of-function mutations were localized to domain-domain interfaces. Our results demonstrate a novel use of macromolecular complex structures for predicting the disease-causing mechanisms through inheritance modes.

## Introduction

Recent advances in high-throughput sequencing technologies enable us to comprehensively identify candidate mutations associated with particular diseases in humans^1^. However, the mechanisms of how the mutations cause the disease are still elusive in many cases. Recent efforts of large cohort projects of whole exome or genome sequencings have revealed that several mutations suggested to be pathogenic were free-riding rare variants and not involved in disease causality^2, 3^. This indicates that we need further evidence, probably based on molecular mechanisms, to convincingly judge the pathogenicity of the genetic variants observed in individuals.

Even though a number of studies have been done to predict the functional or structural impacts of missense mutations^4–6^, the predictions of phenotypic impacts through inheritance modes of the missense mutations are not straightforward. Recessive and dominant are the two major inheritance modes of phenotypes caused by mutations. Most of the recessive mutations result in loss-of-function conditions. On the other hand, the cases of dominant mutations are more complicated than recessive ones, and the mutations are categorized by their molecular mechanisms: haploinsufficiency (HI), dominant-negative (DN), or gain-of-function (GF, including toxic gain-of-function and constitutive activation)^7^. Identifying candidate mutations for dominant diseases is usually much more difficult than identifying recessive ones because the number of deleterious mutations in the heterozygous state (an estimated 50–100 variants in each genome) is usually much higher than that in the homozygous state in each individual^8^.

Most proteins perform their functions by forming particular three-dimensional structures and interacting with other molecules, and pathogenic mutations affect functions by compromising these structures and interactions, as demonstrated by many previous studies^9–16^. In general, pathogenic mutations are frequently observed in sites buried in the interior of a protein molecule or ones involved in macromolecular interactions with a drastic change of amino acid physicochemical properties as compared with harmless non-synonymous substitutions^9–13, 16^. These mutations often destabilize protein structures and/or affect the binding energy to interacting molecules^14, 15^. Thus, protein structures should be very informative in delineating the effects of pathogenic mutations in different inheritance modes and understanding the underlying mechanisms of particular diseases.

The preceding studies have revealed, as an overview, that recessive mutations are mainly biased toward the buried region of protein structures, whereas dominant mutations are significantly enriched in the interfaces of molecular interactions^17, 18^. For example, Zhong *et al*. analyzed 35,154 pathogenic mutations of 1,777 gene-disease pairs and found that in-frame mutations (including not only missense but also small insertions/deletions) associated with autosomal-dominant (AD) diseases likely affect exposed residues on the molecular surface^17^. Guo *et al*. showed that recessive mutations affecting the interface of two interacting proteins tend to cause the same disease whereas dominant mutations do not by investigating 38,497 pathogenic mutations from 1,794 gene-disease pairs^18^.

However, it still remains unclear how the different types of disease-causing mechanisms of dominant mutations (HI, DN, or GF) are associated with the molecular interactions of the mutated residues. This is largely due to a lack of integrated information about the disease mechanisms of each mutation. In this study, we analyzed updated and well-annotated effects of pathogenic mutations on the models of supramolecules with emphasis on the dominant phenotypes, in order to further elucidate the relationship between macromolecular structures and pathogenic mutations.

## Results and Discussion

We first collected the information of missense mutations from the Online Mendelian Inheritance of Man (OMIM) database^19^ and published literature and associated them with phenotypes. A total of 2,512 gene-disease pairs (1,951 unique genes and 2,366 unique diseases) with autosomal recessive (AR) or autosomal dominant (AD) inheritance modes were tabulated (Table 1 and Supplementary Table 1). Further, for 404 out of the 1,140 AD diseases, the types of disease-causing mechanism, namely HI, DN, or GF, of the missense mutations were manually assigned (Supplementary Table 2). Finally, a total of 22,004 pathogenic missense mutation sites were mapped on the protein 3D structures for which known molecular interactions are annotated^20, 21^. Of those residues, 14,164 were assigned to the ordered (structure-determined) regions. The non-synonymous substitutions observed in healthy individuals, denoted as single nucleotide variants (SNV) obtained from the ExAC database^2^ without any cutoff value for allele frequency, were also mapped on the structures, and the locational distributions were compared with the pathogenic missense mutations.

**Table 1:** Statistics of pathogenic and benign mutations.

Category	Genes*	Phenotypes	Mutations	Mapped in 3D	% mapped
All pathogenic mutations	1951	2,366	22004	14164	64.4
Recessive	1233	1372	12340	8271	67.0
Dominant	845	1140	9664	5893	61.0
Haploinsufficiency	115	122	2040	1078	52.8
Dominant-negative	132	143	1724	1129	65.5
Gain-of-function	118	136	985	604	61.8
Others	578	738	4923	3082	62.6
Benign SNV	1782	—	15728	5723	36.4

*Some of the genes appear in two or more categories.

**Figure 1:**
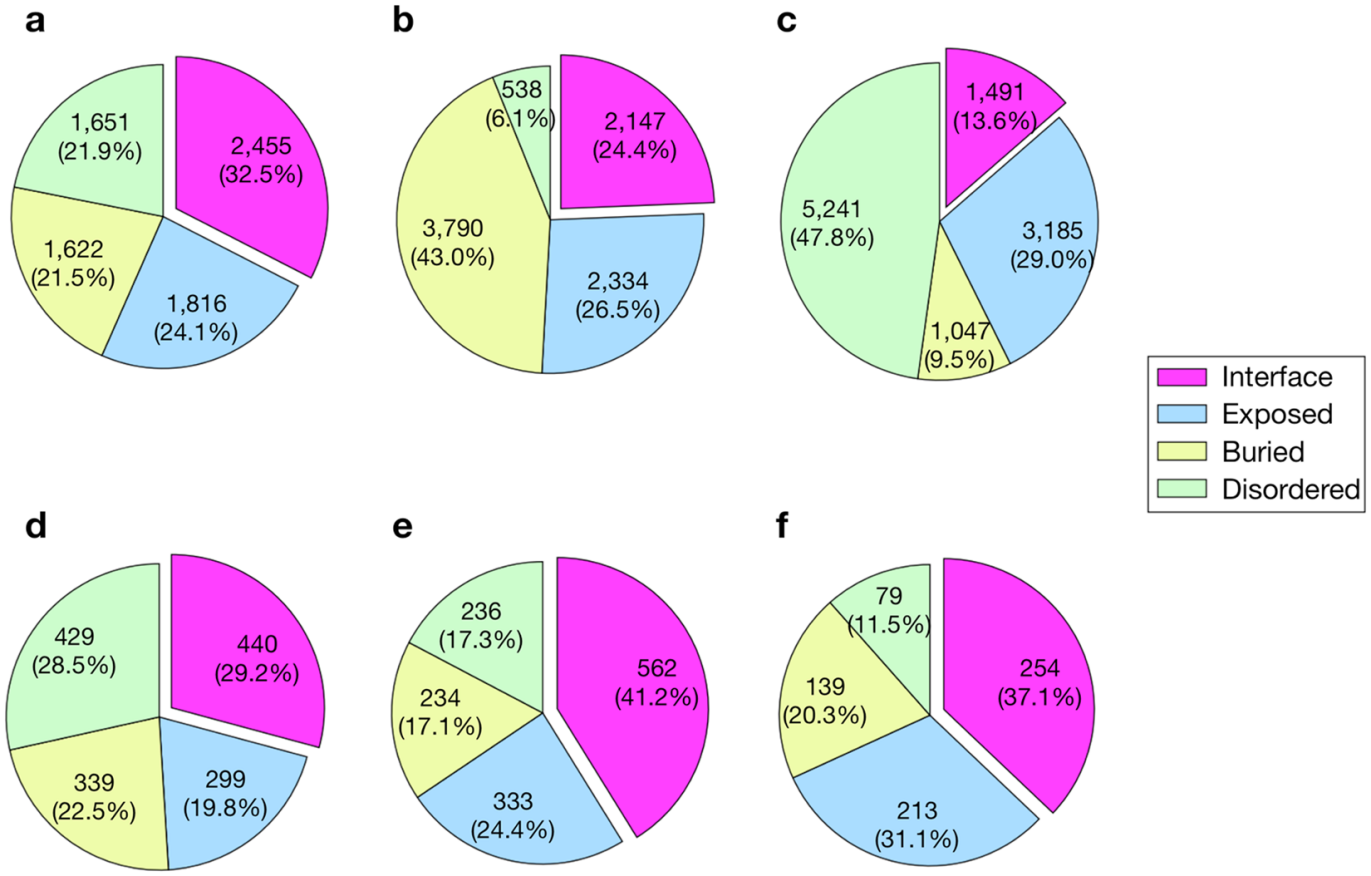
Locational distribution of mutation sites on protein structures. (**a**–**f**) Pie charts represent the distributions of missense mutations on the different structural regions, namely, molecular interface, buried, exposed, or disordered regions, for AD (**a**), AR (**b**), SNV (**c**), HI (**d**), DN (**e**), and GF (**f**).

The results demonstrated that the AR and AD mutations assigned to the ordered regions were significantly higher in number than SNV, nearly half (47.8%) of which were assigned to the intrinsically disordered regions (Fig. 1). When the cutoff value of 1% minor allele frequency (threshold for SNP) was employed for the SNV, *i.e*. a total of 12,215 SNVs with lower frequency were excluded, the proportion of residues located in the disordered regions was slightly increased to 49.3%, whereas those located in the molecular interface was slightly decreased (13.6% to 12.7%). This implies that the non-synonymous substitutions, which were not associated with diseases, tended to avoid regions important for structure formation or molecular interactions, as also reported in previous studies^12, 16–22^.

The locational distributions of AR and AD mutations on 3D structures were predominantly different. For AR mutations, 43.0% were located in the buried region, whereas the same value for AD mutations was only 21.5%. The AD mutations were rather enriched in the residues located in molecular interfaces of the protein-protein or protein-DNA complexes.

Then we compared the structural features of the HI, DN, and GF dominant phenotypes (Fig. 1d-f). The DN and GF but not HI mutations were relatively enriched in the molecular interfaces. More HI mutations were located in the buried regions than expected as compared to those of DN and GF mutations (Supplementary Fig. 1), indicating that the structural localization of HI mutations was similar to that of AR mutations. The AR mutations are thought to cause loss-of-function of the gene products through destabilizing the protein structure, and the result suggested that the underlying molecular mechanism would be similar in the HI phenotypes.

**Figure 2:**
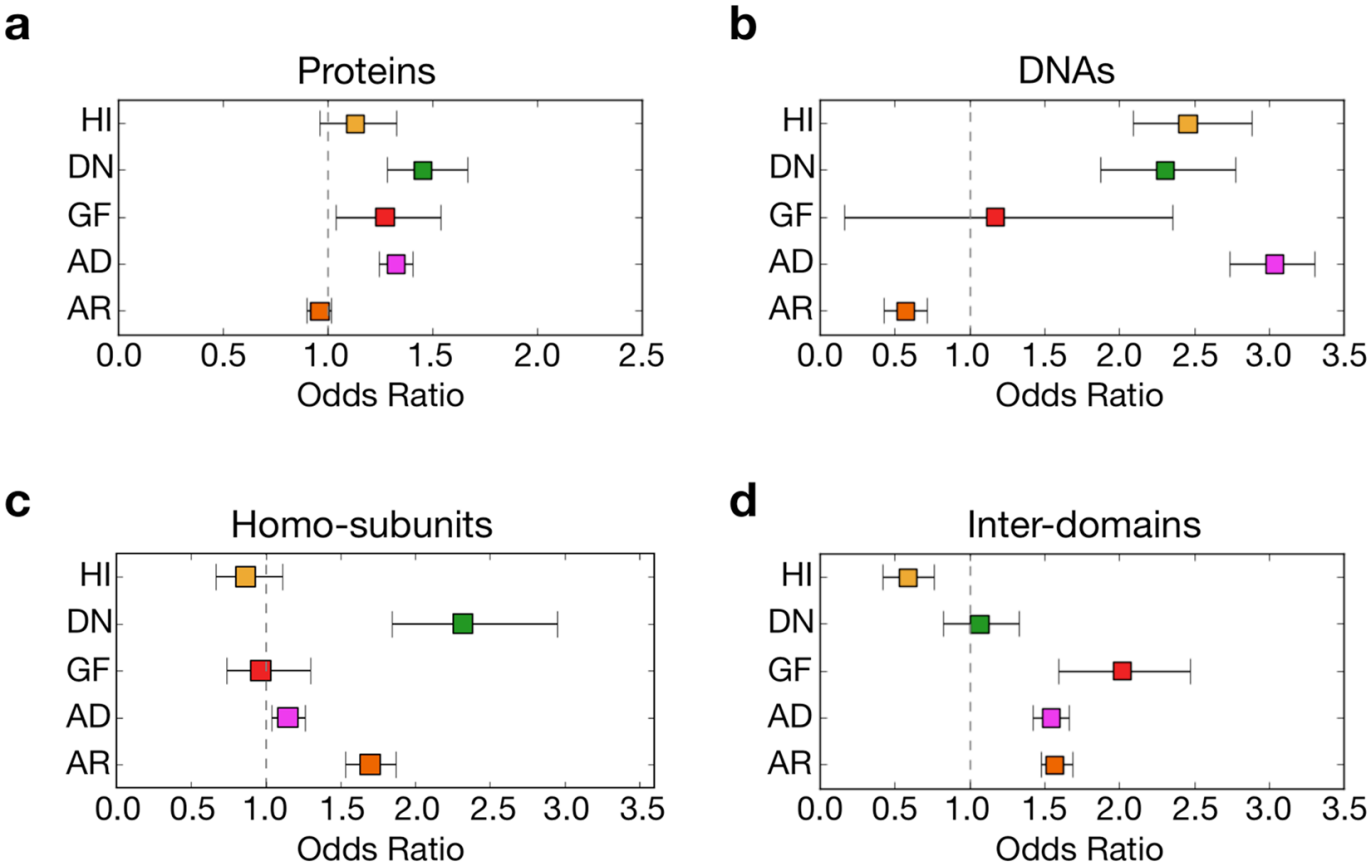
Odds ratio of mutations on interfaces for each inheritance mode. (**a**–**d**) The odds ratio distributions of probabilities that mutations in a category occurred in a given interface of proteins (**a**) or DNA (**b**). (**c**) The ratios of mutations being used for homo over hetero subunit interactions. (**d**) The ratios of mutations used for domain interactions.

The interfaces were classified into those for proteins (PPI) and DNAs (PDI), in order to explore the effects of mutations on molecular interactions in depth (Fig. 2a,b). The AD mutations were biased toward PPI regions [the odds ratio (OR) against random distribution was 1.32, with a 95% confidential interval (CI) of 1.25 to 1.40]. For AR mutations, this trend was the opposite (OR 0.96, 95% CI of 0.90 to 1.02). When the AD mutations were further dissected, the localization of the DN and GF mutations to PPI was rather emphasized (OR 1.45, 95% CI of 1.28 to 1.67 for DN, and OR 1.27, 95% CI of 1.04 to 1.54 for GF), whereas no significant bias was observed for HI (OR 1.13, 95% CI of 0.96 to 1.33). The mutation sites of HI, and DN also, were significantly enriched in PDI (OR 2.46, 95% CI of 2.09 to 2.88 for HI, and OR 2.30, 95% CI of 1.88 to 2.78 for DN) compared to GF (OR 1.17, 95% CI of 0.17 to 2.35).

It is well known that defects in transcription factors often cause diseases by a haploinsufficiency mechanism^23^. Consistently, a large proportion of genes associated with HI (36 genes, 31.3% of total) and DN (26 genes, 19.5%) phenotypes encoded transcription factors in comparison to those with GF phenotypes (6 genes, 5.1%). One possible explanation of why the mutations at DNA-binding sites of transcription factors cause haploinsufficiency is that defects in the DNA-binding ability reflect their own expression levels. Most of such transcription factors would bind to the promoter of their own genes and activate expression.

For example, missense mutations of *GATA2*, encoding a transcription factor protein GATA2, cause familial syndromes with immunodeficiency in a haploinsufficient fashion. GATA2 binds to its own promoter and activates transcription, and the missense mutations at the DNA binding sites impair the promoter binding ability, resulting in decreased transcription of *GATA2* ^24^. Another example would explain dominant-negative mechanisms: mutations in *PITX2*, encoding a homeodomain protein PITX2, are responsible for Axenfeld-Rieger syndrome (MIM# 180500). PITX2 is known to act as a homodimer. Saadi *et al*. demonstrated that the PITX2 mutant protein with the missense mutation Lys88Glu at the DNA binding site formed rather stable heterodimers with the wild-type PITX2 and greatly reduced the binding to the promoter, thus causing disease in a DN manner^25^.

In order to clarify whether the homo or hetero subunit interactions contribute to the difference in disease-causing mechanisms, the PPI interfaces were further divided into those of homo and hetero subunits (Fig. 2c). The AD mutations were slightly biased toward the homo interfaces (the OR of mutations observed in homo interfaces to those in hetero interfaces against the random distribution was 1.15, 95% CI of 1.04 to 1.26). The DN mutations tended to be on homo interfaces (OR 2.58, 95% CI of 2.05 to 3.28), whereas no significant bias between homo and hetero subunit interfaces was observed for HI and GF mutations.

The Gene Ontology enrichment analysis showed that a GO term of “protein homooligomerization” was significantly enriched in the genes associated with DN phenotypes (12 genes, *p = *9.6 × 10^−8^) as compared to those with HI (no genes) or GF (3 genes, *p* > 0.05) phenotypes, suggesting that proteins which act as homooligomers are highly associated with DN phenotypes. Although the total numbers of residues for the homo subunit interfaces (6,865 residues) were not significantly different from those for the hetero subunit interfaces (6,174 residues) in the proteins with DN phenotypes, the mutations were significantly enriched (*p = *8.7 × 10^−7^ by binomial test) in the homo (283 residues) compared to the hetero (110 residues) subunit interfaces in the same proteins. The mutations in the homo subunit interfaces might disrupt appropriate oligomer formation or contribute to formation of an inactive oligomer in the dominant-negative manner, as previously suggested^26^.

We further investigated the structural features of the missense mutations in terms of domain–domain interactions in a single protein molecule. Domain configurations are also important in regulating protein functions, and some of the residues not involved in inter-molecular interactions are used for inter-domain interactions. The ECOD database was referred to in determining domain boundaries, and some of the mutant sites, which were assigned to buried or non-interface regions, were reassigned to the domain interface. Interestingly, the GF mutations were enriched in the domain–domain interfaces (the OR against random interaction was 2.0, 95% CI of 1.6 to 2.5) compared to those with HI and DN mutations (Fig. 2d). This value of AD mutations (OR 1.5, 95% CI of 1.4 to 1.7) was comparable to those with AR mutations (OR 1.6, 95% CI of 1.5 to 1.7). The result suggested that one major cause of a gain-of-function phenotype would be a mutation that compromises the regulatory function accomplished through the inter-domain interaction within a single protein molecule.

**Figure 3:**
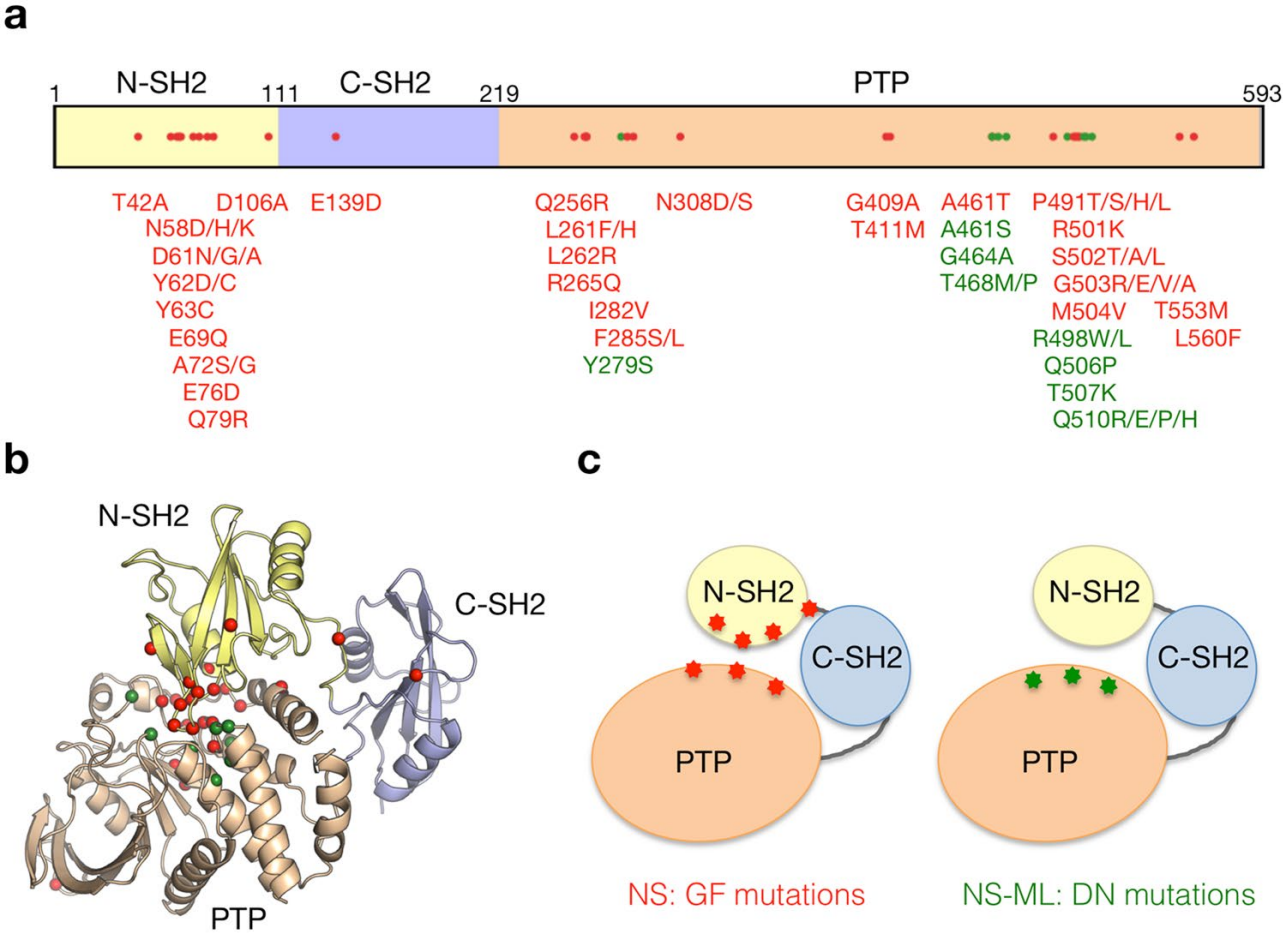
Locational distribution of mutation sites on PTPN11 protein. (**a**) The schematic primary structure of PTPN11 with structural domains and pathogenic missense mutations. The mutations associated with different inheritance modes are colored differently (DN and GF mutations are shown in green and red, respectively). (**b**) Mapping of the mutations on the crystal structure of human PTPN11 (PDB code: 2shp). The mutant residues are represented by sphere models. (**c**) The schematic models of the PTPN11 domain interaction with pathogenic mutations.

For example, the GF mutations of *PTPN11* encoding SH2 domain-containing protein-tyrosine phosphatase 2 (SHP2), which are responsible for Noonan syndrome (NS, MIM #163950), were mainly located in the interface between N-SH2 and PTP domains (Fig. 3). These mutations altered the residues involved in the autoinhibitory interaction, thus made the molecule easily assume the open conformation, and consequently increased the constitutive catalytic activity of the phosphatase^27^. On the other hand, the reported DN mutations of SHP2 were located only in the PTP domain and were closed to the substrate binding or catalytic sites of SHP2. These mutations were demonstrated to decrease the phosphatase activity, resulting in Noonan syndrome with multiple lentigines (NS-ML, MIM #151100, formerly called LEOPARD syndrome) due to the DN effect^28^.

Another example, perhaps the most appropriate to present the results of this study, is the case of *STAT1*, which encodes a transcription factor STAT1 that regulates the development of various types of immune cells. Defects of *STAT1* cause several diseases, e.g. the autosomal recessive STAT1 deficiency (AR-STAT1 deficiency, MIM #613796), the autosomal dominant STAT1 deficiency (AD-STAT1 deficiency, MIM #614162), and the autosomal dominant chronic mucocutaneous candidiasis (CMC, MIM #614892).

**Figure 4:**
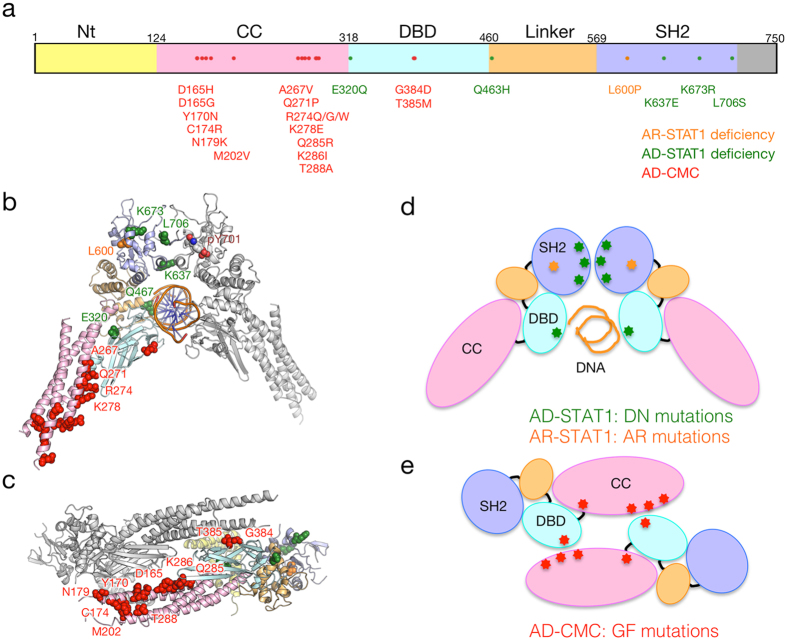
Locational distribution of mutation sites on STAT1 protein. (**a**) The schematic primary structure of STAT1 with structural domains and pathogenic missense mutations. (**b,c**) The mutations associated with different inheritance modes are colored differently. Mappings of the mutations on the crystal structures are shown for the parallel dimer (**b**) PDB code: 1bg1) and anti-parallel dimer (**c**) PDB code: 1yvl). The domains are colored according to panel a. The mutant residues are represented by sphere models. (**d,e**) The schematic models of the parallel dimer (**d**) and anti-parallel dimer (**e**) with the pathogenic mutations with phenotypes. The mutation associated with AR-STAT1 deficiency (colored orange) was buried in the SH2 domain.

The known disease-causing mutations were mapped on the crystal structure of human STAT1 complexes (Fig. 4b,c). The missense mutation Leu600Pro, which is known to cause the AR-STAT1 deficiency, was located in the buried region of the SH2 domain (Fig. 4b,d). The mutation, hence, was suggested to destabilize the SH2 domain leading to reduction of the protein expression level by degradation. The mutations associated with the AD-STAT1 deficiency, which is known to occur in DN mode^29, 30^, were located in the homo-dimer interface of SH2 domain or DNA-binding domain (DBD) (Fig. 4b). The DN mutations impaired STAT1 phosphorylation and DNA-binding activity^29^. Contrary to the AD-STAT1 deficiency, CMC is caused by GF mode^31, 32^. The CMC mutations showed increased phosphorylation of Tyr701 of STAT1 due to impaired nuclear dephosphorylation. The mutated residues were mainly located in the coiled-coil domain (CCD), which was thought to work in the dephosphorylation of STAT1 by forming an anti-parallel dimer (Fig. 4c). Most of the GF (CMC) mutations were located in this interface (Fig. 4d), and they would disrupt the formation of the anti-parallel dimer. A couple of GF (CMC) mutations were, however, not observed in this interface, but were located in the domain interface. For example, one of the GF (CMC) mutations, Arg274Gln, was found in the interface between the CCD and DBD. Recently, it was demonstrated that the Arg274 was involved in the regulation of STAT1 activity through the interaction with the DBD^33^.

## Conclusion

In summary, we demonstrated a clear correlation between the inheritance modes of missense mutations and protein 3D structures. A major structural location of AR mutations is the buried region of a protein, and the mutations cause destabilization of the protein structure. In AD mutations, the structural locations varied depending on the mechanisms of dominant inheritance modes; DN mutations are biased toward molecular interfaces and affect interactions with the cognate (homo) protein or DNA (Figs 3c and 4d,e). HI mutations are enriched on the interface with DNA and buried regions. GF mutations, as a unique tendency, affect domain-domain interactions, which often resulted in constitutive activation of proteins. Our findings would be useful in precisely predicting the pathogenic mutations responsible for dominant diseases based on the molecular basis, especially those of gain-of-function cases, which are currently regarded as challenging^34^ for evaluating the pathogenicity of mutations in clinical diagnosis.

## Methods

### Constructing disease datasets

The data on the relationship between genes and their associated diseases in humans were retrieved from OMIM (http://omim.org/) using OMIM API and saved as XML format. The human genes associated with at least one phenotype with an autosomal recessive (AR) or dominant (AD) inheritance mode were selected. The association information between OMIM Gene and NCBI Gene was extracted from a mim2gene.txt file obtained from the OMIM database. The genes with no link to a RefSeq entry were excluded.

For each dominant disease, the molecular mechanisms of causality by mutations, namely haploinsufficiency (HI), dominant-negative (DN), or gain-of-function (GF) were manually extracted from the OMIM XML file. If a notation “haploinsufficiency” was included in the description of the disease in the XML file, the disease was considered as HI. When “dominant-negative” or “dominant negative” was noted, the disease was considered as DN. For GF, “gain-of-function”, “toxic gain of function”, “activating mutation,” or “constitutively active” was employed for the discrimination key.

### Obtaining missense mutation data

The disease-associated missense mutations in humans were obtained from ClinVar^35^ (as of Aug. 7, 2016) and HGMD^36^ professional^®^ (as of August 2016). Because the databases also contained non-pathogenic variant entries, the entries were further selected by referring to a significance code, “Pathogenic” or “Likely pathogenic” for ClinVar, or the variant tag “DM” for HGMD. The mutation data not associated with an OMIM phenotype number were discarded.

### Assignment of 3D structural positions of mutations

The amino acid sequences of RefSeq^37^. Entries for the disease-causing genes were subjected to a search for the homologous amino acid sequences of the PDB^21^ entries (as of August 2016) using the BLAST + program^38^ with a cutoff E-value of 10^−4^. When the sequence identity between the target and the protein with 3D structure was > 30%, the amino acid residue sites of the target protein were assigned according to the sequence alignment between the two sequences.

Amino acid residues in the interfaces of molecular interactions were extracted according to the biological assembly structures of PDB. Structural domain data were obtained from the ECOD database^39^. If at least one atom of the amino acid residue was within 4.5 Å of the residues in other chains (either protein or DNA) or domains, the residues were assigned to the molecular interface or domain interface. Intrinsically disordered regions of the amino acid sequences were predicted by DISOPRED ver. 3.1^40^.

### Statistical analysis of missense mutations

The fraction of mutations for a given disease category *i* located in a type of interface *j* (*p*_*ij*_) was calculated by dividing the sum of the mutation sites for the category observed on the type of interfaces by the total number of mutations located in the same type of interface. We calculated the expected fraction for a type of interface *j* (q_*ij*_) by dividing the sum of the residues located in the type of interface by the sequence length of all proteins. The odds ratios (ORs) were calculated on the bases of the observed and expected fractions as

$${\rm{OR}}={p}_{ij}/(1-{p}_{ij})/{q}_{ij}/(1-{q}_{ij})$$

In order to estimate the 95% confidence intervals of each OR, 1,000 bootstrap replicates were generated for mutations in each disease category.

### Gene ontology enrichment analysis

The gene ontology (GO) terms annotated with the human disease-causing genes were extracted from the NCBI Gene database. For gene sets in each category, the hypergeometric test originally described by Draghici *et al*.^41^ was applied with a correction for multiple testing using the false discovery rate (FDR). The GO terms with a FDR < 0.05 were selected as functionally enriched terms.

## Electronic supplementary material

Supplementary Information

Supplementary Table 1

Supplementary Table 2
