# Supplementary material for: Decoding disease-causing mechanisms of missense mutations from supramolecular structures
Source: Sci Rep. 2017 Aug 17;7:8541. doi: 10.1038/s41598-017-08902-1 (PMC5561164; doi:10.1038/s41598-017-08902-1)
Supplement: Supplementary file 1 — Supplementary Information [file 41598_2017_8902_MOESM1_ESM.doc]

**Supplementary Information: Decoding disease-causing mechanisms of missense mutations from supramolecular structures**

Atsushi Hijikata^1^, Toshiyuki Tsuji^1,2^, Masafumi Shionyu^1^, Tsuyoshi Shirai^1,*^

1. Faculty of Bioscience, Nagahama Institute of Bio-Science and Technology, 1266 Tamura-cho, Nagahama, Shiga 526-0829, Japan
2. MITA International School, Yoga, Setagaya, Tokyo, Japan

*To whom correspondence should be addressed: Tsuyoshi Shirai (t_shirai@nagahama-i-bio.ac.jp)


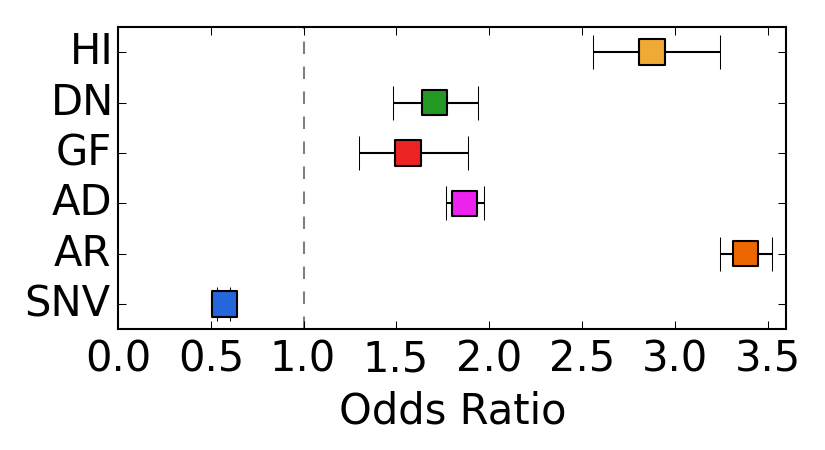


**Supplementary Figure 1.**

**Odds ratio of mutations in buried region for each inheritance mode.** The odds ratio distributions of probabilities that mutations in a category occurred in buried regions of 3D structures.

**Supplementary Tables (Excel)**

**Supplementary Table 1.** A list of the genes-disease pairs analyzed in this study. This file includes the RefSeq accession, amino acid length, MIM numbers and the mode of inheritances for each gene.

**Supplementary Table 2.** A list of the genes associated with the different dominant inheritance modes. The gene list for each mode is separated in different sheets, HI for haploinsufficiency, DN for dominant-negative and GF for gain-of-function. This file includes the RefSeq accession, amino acid length, MIM numbers, and the mode of inheritance for each gene.
